# Prediction of neoadjuvant chemotherapy pathological complete response for breast cancer based on radiomics nomogram of intratumoral and derived tissue

**DOI:** 10.1186/s12880-024-01198-4

**Published:** 2024-01-20

**Authors:** Guangying Zheng, Jie Hou, Zhenyu Shu, Jiaxuan Peng, Lu Han, Zhongyu Yuan, Xiaodong He, Xiangyang Gong

**Affiliations:** 1 Jinzhou Medical University, Jinzhou, Liaoning Province China; 2 Cancer Center, Department of Radiology, Zhejiang Provincial People’s Hospital (Affiliated People’s Hospital), Hangzhou Medical College, No. 158 Shangtang Road, Hangzhou City, Zhejiang Province China

**Keywords:** Radiomics, Background parenchymal enhancement, Breast cancer, Neoadjuvant chemotherapy, Pathological complete response

## Abstract

**Background:**

Non-invasive identification of breast cancer (BCa) patients with pathological complete response (pCR) after neoadjuvant chemotherapy (NACT) is critical to determine appropriate surgical strategies and guide the resection range of tumor. This study aimed to examine the effectiveness of a nomogram created by combining radiomics signatures from both intratumoral and derived tissues with clinical characteristics for predicting pCR after NACT.

**Methods:**

The clinical data of 133 BCa patients were analyzed retrospectively and divided into training and validation sets. The radiomics features for Intratumoral, peritumoral, and background parenchymal enhancement (BPE) in the training set were dimensionalized. Logistic regression analysis was used to select the optimal feature set, and a radiomics signature was constructed using a decision tree. The signature was combined with clinical features to build joint models and generate nomograms. The area under curve (AUC) value of receiver operating characteristic (ROC) curve was then used to assess the performance of the nomogram and independent predictors.

**Results:**

Among single region, intratumoral had the best predictive value. The diagnostic performance of the intratumoral improved after adding the BPE features. The AUC values of the radiomics signature were 0.822 and 0.82 in the training and validation sets. Multivariate logistic regression analysis revealed that age, ER, PR, Ki-67, and radiomics signature were independent predictors of pCR in constructing a nomogram. The AUC of the nomogram in the training and validation sets were 0.947 and 0.933. The DeLong test showed that the nomogram had statistically significant differences compared to other independent predictors in both the training and validation sets (*P* < 0.05).

**Conclusion:**

BPE has value in predicting the efficacy of neoadjuvant chemotherapy, thereby revealing the potential impact of tumor growth environment on the efficacy of neoadjuvant chemotherapy.

**Supplementary Information:**

The online version contains supplementary material available at 10.1186/s12880-024-01198-4.

## Introduction

Breast cancer (BCa) is a common malignancy among women and one of the deadliest cancers in the world [1, 2]. Neoadjuvant chemotherapy (NACT) is a crucial strategy for treating BCa, but its efficacy varies significantly among patients, with some patients achieving pathologic complete response (pCR) while others do not. In contrast, BCa patients with pCR experience significantly longer disease-free survival and overall survival than those without pCR [3]. Therefore, predicting pCR is significant for developing personalized treatment plans [4].

Radiomics is an emerging interdisciplinary field that combines imaging and computer science to comprehensively analyze tumors from multiple aspects such as morphology, metabolism, blood flow, and intensity, to predict patient treatment response and prognosis [5]. Traditional radiomics analysis focuses on primary tumor lesions, as their histological type is closely related to the patient’s treatment response and prognosis [6]. However, In recent years, tumor-adjacent tissue has also received increasing attention. Tumor adjacent tissue refers to normal tissue within a certain range around a tumor, whose interactions with the tumor may affect its growth, invasion, and treatment response [7]. Earlier investigations have demonstrated that the radiomics features extracted from peritumoral tissue can also predict pCR for BCa [8, 9]. In addition, normal breast tissue outside the tumor cannot be ignored, among which background parenchymal enhancement (BPE) refers to the enhancement degree and distribution characteristics of normal breast tissue outside the tumor in enhanced MRI images, which are influenced by various factors such as hormone levels, age, and breast tissue density [10]. New research has indicated that BPE is strongly tied to the occurrence and prognosis of BCa [11]. Hence, the evaluation of BPE is one of the key components of assessing the efficacy of NACT for BCa [12]. However, previous studies have focused on the BPE of the contralateral breast. Tumor growth can have physiological vascularization and perfusion effects on the ipsilateral breast tissue, thereby affecting the determination of BPE [13]. Conversely, considering the primary tumor tissue, radiomics may better reflect the changes within the tumor tissue rather than measuring it through conventional vascular enhancement intensity [14]. Therefore, we hypothesize that radiomics feature extracted from BPE tissue on the same side of the primary tumor can better predict pCR in patients with BCa. Combining the radiomics features of the Intratumoral and peritumoral may further improve predictive efficiency.

Our primary objective is to explore the correlation between various tissue radiomics features and treatment response, to identify specific features to construct radiomics signature for predicting pCR in NACT. Secondly, we will use a combination of clinical features and radiomics signature to develop a highly accurate prediction model using machine learning algorithm and compare its predictive effectiveness with clinical indicators.

## Materials and methods

### Patient information

**Fig. 1:**
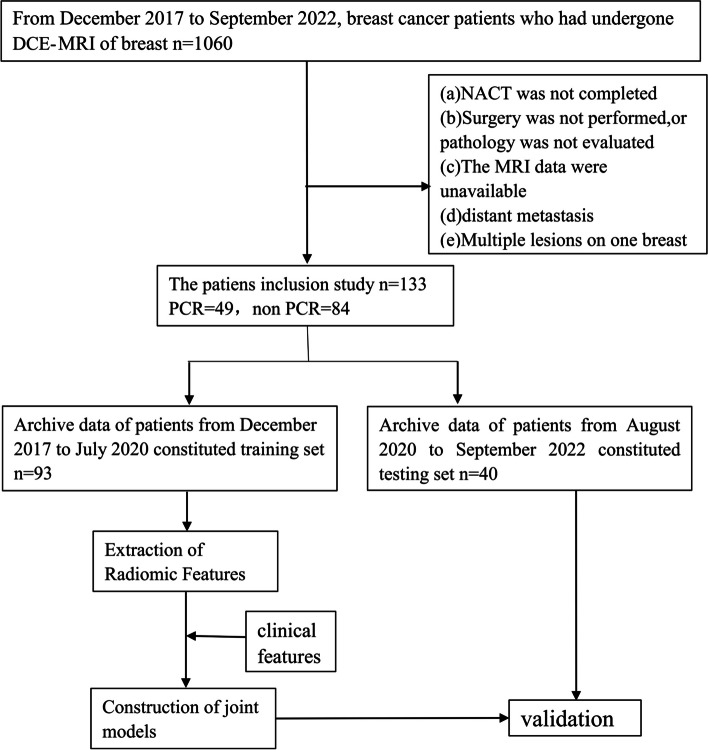
Flowchart showed the recruitment of patients and the overall design of this retrospective study

This retrospective study was approved by the Ethics Committee of Zhejiang Provincial People’s Hospital (No. QT2023380). From December 2017 to September 2022, A total of 1060 patients with BCa were diagnosed through the Picture Archiving and Communication System (PACS), and 133 patients were eventually enrolled based on the following inclusion criteria: (a) non-specific invasive breast cancer confirmed by pre-NACT biopsy; (b) receive standard 6–8 cycles of NACT without prior treatment history; (c) surgery is performed after completion of NACT, and pCR is confirmed based on the postoperative pathological evaluation. Exclusion criteria for this study were as follows: (a) patients did not complete NACT or received non-standard treatment; (b) No surgery was performed after NACT or surgery was performed in an outside hospital, and postoperative pathology was not evaluated; (c) poor MRI image quality (e.g., artifacts); (d) multiple lesions on one breast; (e) distant organ metastasis. In addition, patients diagnosed between December 2017 and July 2020 were grouped into a training set (*n* = 93) to build the model, while patients diagnosed between August 2020 and September 2022 were grouped into a validation set (*n* = 40) to verify the reliability of the model. The recruitment path of subjects and the study design for this study are shown in the Fig. 1.

### Image preprocessing and segmentation

All breast dynamic contrast-enhanced MRI scans were performed using the 3.0 Tesla MRI scanner (Skyra; Siemens Healthineers). The imaging protocol and detailed parameters are provided in the Supplementary materials and Table S1. Before feature extraction, T1WI was adopted as a rigid alignment template for all sequences using the alignment function of the SPM toolkit in Matlab software. T1-weighted imaging (T1WI), enhanced third-phase T1-weighted (T1 + C) sequences, and dynamic contrast-enhanced subtraction images were preprocessed and aligned to ensure the three sequences contained the same resolution, spacing, and origin. This was done to reduce the potential influence of scan protocol parameters. The normalized T1WI images were imported into ITK software, and the entire tumor area, peritumoral area, and BPE area were segmented layer by layer to determine the volume of interest (VOI). Finally, the VOI of these three regions were subjected to feature extraction using the Pyradiomics program, respectively. Depending on the alignment of the sequence, T1WI, T1 + C and dynamic contrast-enhanced subtraction images can share the same VOI from T1WI to extract features.

### Clinical and radiological variables

All patients were biopsied pre-NACT. Based on biopsy results, we evaluated the expression of several receptors and antigens, including progesterone receptor (PR), estrogen receptor (ER), antigen Ki67 (Ki-67), and human epidermal growth factor receptor 2 (HER2). Nuclear staining of ER and PR ≥ 1% indicates positive. Then Ki-67 expression value > 20% was positive. For HER2 expression, an IHC score equal to 0 or 1 + is considered negative, and 3 + is considered HER2 positive. An IHC score equal to 2 + requires in situ hybridization (ISH), and the structure shows non-amplification for HER2-negative and amplification for HER2-positive.

Based on the expression of ER, PR, Ki-67, and HER2, we classified all BCa patients into 5 subtypes, including Luminal A, Luminal B HER2 negative, Luminal B HER2 positive, HER2 positive non-luminal, and triple negative. Specific standards refer to the St Gallen International Expert Consensus [15].

All patients histopathological examinations and analyses are performed by professional breast pathologists with more than 10 years of experience in the field of breast pathology. They were blind to MRI data, and all specimens were evaluated using the Miller-Payne system [16]. The Miller-Payne system has five levels. pCR is defined as the absence of residual invasive cancer in the specimen (possible presence of residual ductal carcinoma in situ). Moreover, during axillary lymph node dissection, there was no lymph node invasion (yPT0/isN0) in ipsilateral sentinel lymph nodes or resected lymph nodes. For additional details on pathology grading, please refer to the Supplementary materials.

### Extraction of radiomic features

A total of 3396 radiomic features were extracted from each of the three sequences, which encompassed six categories of features, namely shape, first-order, Gray Level Concurrence Matrix (GLCM), Gray-Level Run-Length Matrix (GLRLM), gray-level size zone matrix (GLSZM), and Gray level co-occurrence matrix (GLDM). Three sequences were scanned in one patient, providing 10188 radiomic features per patient. Moreover, to ensure the accuracy and stability of the radiomic features, two radiologists (radiologist A and radiologist B) manually segmented the tumors using ITK software, resulting in two sets of features, set A and set B. The Spearman’s rank correlation test was then performed to evaluate the correlation coefficient (CC) of each feature between the two sets. Features with CC > 0.8 were deemed robust.

### Establishment of an optimal radiomics signature based on machine learning

To exclude non-repeatable, redundant and irrelevant features from the extracted features of the initial set, we performed feature dimensionality reduction using mRMR (maximum relevance and minimum redundancy) and GBDT (gradient boosted decision number) ensemble dimensionality reduction methods for the set of radiomics features extracted from the training set. Then, we used logistic regression to construct intratumoral, BPE, and intratumoral + BPE models and used decision tree algorithms in machine learning to construct radiomics signatures based on the optimal model. The score value of each case calculated based on this signature reflects the probability of pCR, and these score values are named rad-score. To assess the performance of the radiomics signature, we plotted receiver operating characteristic (ROC) curves and compared the probability difference between pCR and non-pCR.

### Construction and validation of joint models

**Fig. 2:**
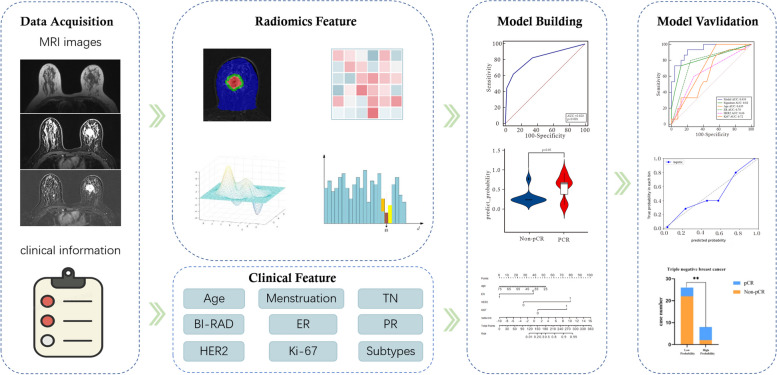
Research flowchart for model construction and validation

Multivariate logistic regression analysis and backward stepwise selection method with the stopping rule, as specified by Akaike’s information criterion (AIC), were conducted to select independent predictors from age, clinical TN stage, menopausal status, BI-RAD stage, ER, PR, HER2, Ki-67, and radiomics signature, based on which a joint model was built and a nomogram was generated. The model’s goodness of fit was assessed using the Hosmer–Lemeshow test, and the agreement between the predicted and actual pCR probability was evaluated using calibration curves. To validate the improvement in model performance after including the radiomics signature, we used area under curve (AUC) to assess the performance of the combined model and independent predictors and the DeLong test to determine the difference between the combined model and other independent predictors. Furthermore, we extended the assessment of the model’s clinical efficacy by calculating the probability of pCR in each Luminal type using the combined model, as pCR predictive effect can be unstable in triple-negative BCa patients. Finally, the optimal cutoff value corresponding to the Yuden index of the ROC curve was used as a threshold to divide the pathology in each Luminal fraction into a low and a high probability group, and their pathological pCR results were compared. The entire research process is illustrated in Fig. 2.

### Statistical analysis

Statistical analyses were performed with Python (version 3.5), SPSS software (version 24.0) and MedCalc software (version 11.2). The Kolmogorov–Smirnov test was used to test the normality of the data. Normally distributed data were evaluated using an independent-sample t-test, whereas nonnormally distributed data were evaluated using the Mann–Whitney U test. The difference between categorical variables was tested with the chi-squared test. The calculated AUC was used to evaluate the performance of the model for the prediction of pCR. A two-tailed *P* value < 0.05 indicated statistical significance.

## Results

### Comparison of clinical data

There was no statistically significant difference in clinical data between the training and test sets (*P* > 0.05). However, there was a statistical difference in the training and validation sets in ER, PR, HER2, Ki-67 and BCa subtypes between the pCR and non-pCR groups (*P* < 0.05). In addition, the BI-RAD stage in the training set was also statistically different between the pCR and non-pCR groups (*P* < 0.05). At the same time, the rest of the clinical data were not statistically different (*P* > 0.05), as detailed in Table 1.

**Table 1:** Comparison of clinical information in the training and validation sets

Characteristics		Training cohort (*n* = 93)				Validation cohort (*n* = 40)				Training* VS* Validation *P* value
		**ALL cohort**	Non pCR	pCR	***P* value**	**ALL cohort**	Non pCR	pCR	***P* value**	
Age(year)		51.51 ± 10.42	52.20 ± 10.32	49.56 ± 10.08	0.053	48.15 ± 12.08	48.28 ± 13.37	47.93 ± 10.00	0.931	0.107
Menstrual status	Premenopausal	45 (48.39%)	25 (42.37%)	20 (58.82%)	0.126	26 (65.00%)	14 (56.00%)	12 (80.00%)	0.123	0.078
	Postmenopausal	48 (51.61%)	34 (57.63%)	14 (41.14%)		14 (35.00%)	11 (44.00%)	3 (20.00%)		
T stage	T1-2	69 (74.19%)	41 (69.49%)	28(82.35%)	0.172	31 (77.50%)	21 (84.00%)	10 (66.67%)	0.379	0.686
	T3-4	24 (25.81%)	18 (30.15%)	6 (17.65%)		9 (22.50%)	4 (16.00%)	5 (33.33%)		
N stage	N1	21 (22.58%)	14 (23.73%)	7 (20.59%)	0.727	14 (35.00%)	6 (24.00%)	8 (53.33%)	0.06	0.136
	N0	72 (77.42%)	45 (76.27%)	27 (79.41%)		26 (65.00%)	19 (76.00%)	7 (46.67%)		
BI-RAD stage	4	5 (5.38%)	1 (1.69%)	4 (11.76%)	0.024^*^	5 (12.50%)	3 (12.00%)	2 (13.33%)	0.9	0.214
	5	58 (65.90%)	41 (69.49%)	17 (56.67%)		19 (47.50%)	11 (44.00%)	8 (53.33%)		
	6	30(34.09%)	17 (28.81%)	13 (43.33%)		16 (40.00%)	11 (44.00%)	5 (33.33%)		
ER status	Negative	35 (37.63%)	16 (27.12%)	19 (55.88%)	0.006^*^	18 (45.00%)	6 (24.00%)	12 (80.00%)	0.001^*^	0.426
	Positive	58 (62.37%)	43 (72.88%)	15 (44.12%)		22 (55.00%)	19 (76.00%)	3 (20.00%)		
PR status	Negative	39 (41.94%)	19 (32.20%)	20 (58.82%)	0.012^*^	19 (47.50%)	7 (28.00%)	12 (80.00%)	0.001^*^	0.553
	Positive	54 (58.06%)	40 (67.80%)	14 (41.18%)		21 (52.50%)	18 (72.00%)	3 (20.00%)		
HER2 status	Negative	55 (59.14%)	44(74.58%)	11(32.35%)	< 0.001^*^	24 (60.00%)	18 (72.00%)	6 (40.00%)	0.046^*^	0.926
	Positive	38 (40.86%)	15(25.42%)	23(67.65%)		16 (40.00%)	7 (28.00%)	9 (60.00%)		
Ki-67 status	Negative	27 (29.03%)	23 (38.98%)	4 (11.76%)	0.005^*^	11 (27.50%)	11 (44.00%)	0 (0.00%)	0.008^*^	0.858
	Positive	66 (70.97%)	36 (61.02%)	30 (88.24%)		29 (72.50%)	14 (56.00%)	15 (100.00%)		
breast cancer subtypes	Luminal A	10 (10.75%)	10 (16.95%)	0 (0.00%)	< 0.001^*^	2 (5.00%)	2 (8.00%)	0 (0.00%)	0.004^*^	0.137
	Luminal B	50 (53.76%)	33 (55.93%)	17 (50.00%)		20 (50.00%)	17 (68.00%)	3 (20.00%)		
	HER2 positive non luminal	13 (13.98%)	2 (3.39%)	11 (32.35%)		12 (30.00%)	4 (16.00%)	8 (53.33%)		
	triple negative	20 (21.51%)	14 (23.73%)	6 (17.65%)		6 (15.00%)	2 (8.00%)	4 (26.67%)		

*indicates statistical significant difference

### Development of the radiomics signature and assessment of its accuracy

**Fig. 3:**
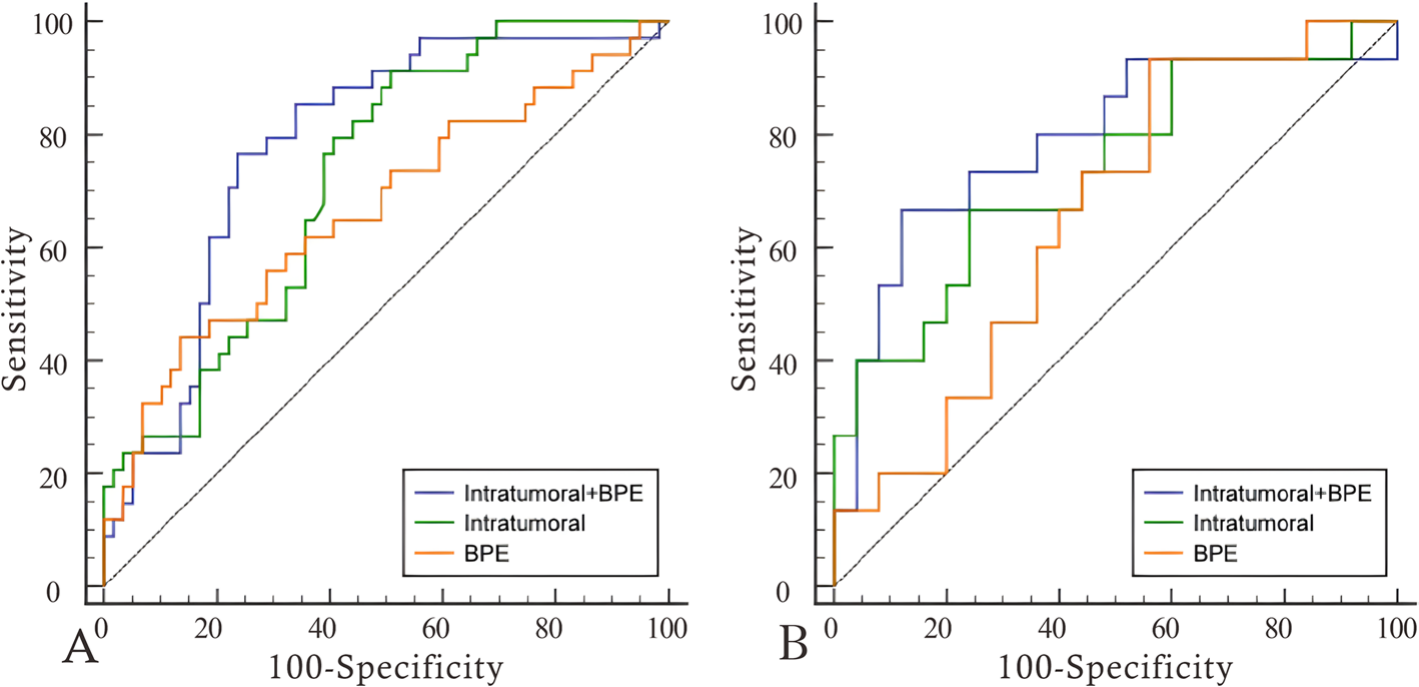
ROC curves of models from intratumoral, BPE and combinations of them in the training set (**A**) and the validation set (**B**)

**Fig. 4:**
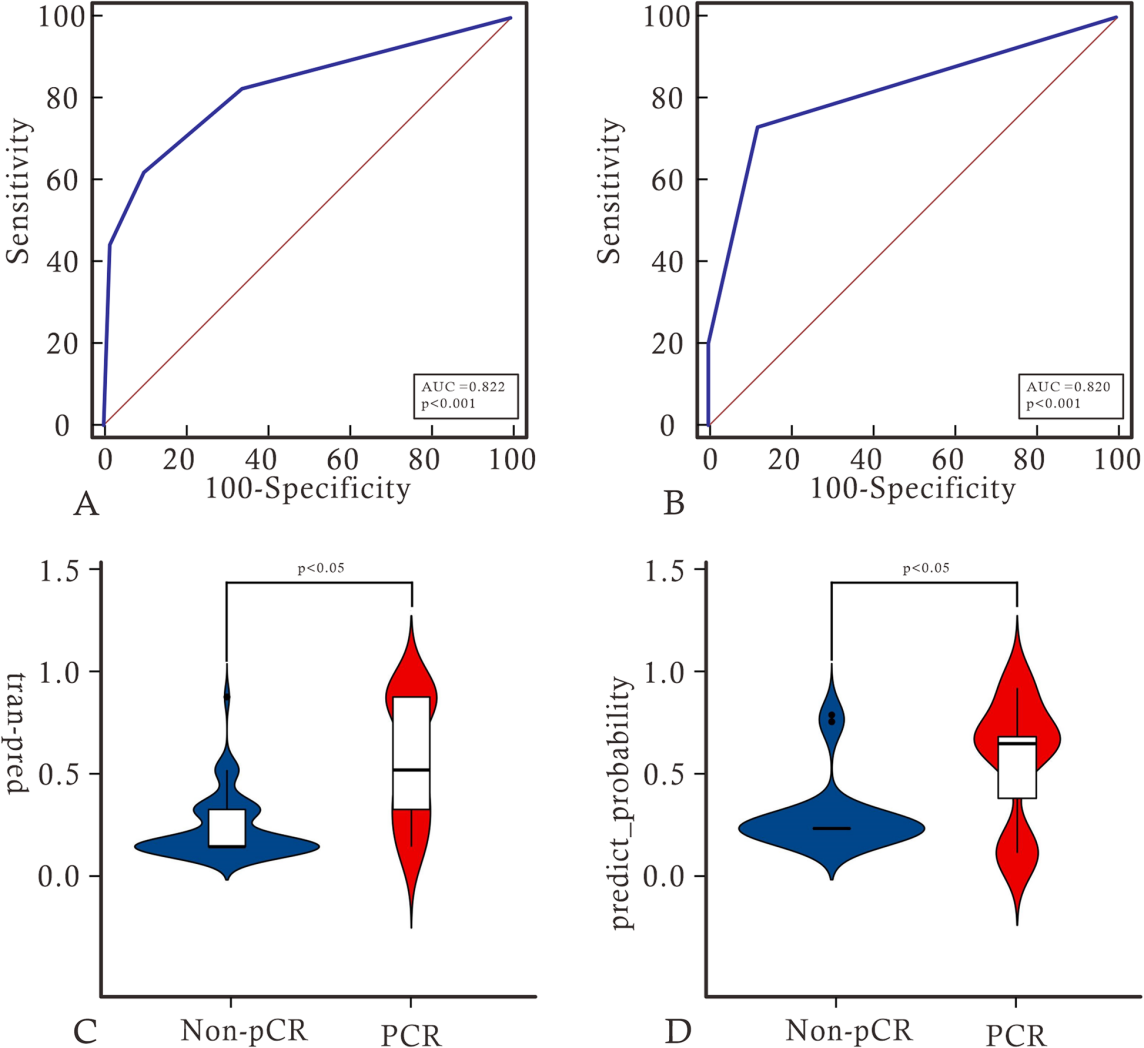
**A** and **B** show the ROC curve analysis of radiomics labels in the training and validation sets. **C** and **D** show the difference violin plots based on label scores in the non-pCR and pCR groups, respectively

We extracted 7 features to construct the radiomics signature, including 2 features from dynamic contrast-enhanced subtraction images and 2 from T1WI images, all from the intratumoral region, as well as 3 features from enhancement images, 1 from the intratumoral region and 2 from the BPE region. Detailed feature information is provided in the Supplementary materials. Using logistic regression to construct intratumoral, BPE, and intratumoral + BPE models. Among signatures for a single region, intratumoral had the best predictive value. The diagnostic performance of the intratumoral improved after adding the BPE signature. radiomics signature was constructed using decision trees based on the optimal model of intratumoral + BPE, and the radiomics signature achieved AUCs of 0.822 and 0.820, sensitivities of 0.618 and 0.733, and specificities of 0.898 and 0.880 in the training and testing groups, respectively. Furthermore, the decision tree scores were statistically different between pCR and non-pCR in the training and validation sets (*P* < 0.05). Please refer to Table 2, Figs. 3 and 4 for further details.

**Table 2:** Diagnostic performance of models from intratumoral, BPE and combinations of them in the training set and the validation set

	Model	AUC (95% CI)	Sensitivity	Specificity
Training	Intratumoral	0.723 (0.621–0.811)	0.912	0.492
	BPE	0.666 (0.561–0.760)	0.441	0.864
	Intratumoral + BPE	0.782 (0.684–0.861)	0.765	0.763
Validation	Intratumoral	0.736 (0.573–0.862)	0.667	0.760
	BPE	0.659 (0.492–0.801)	0.933	0.440
	Intratumoral + BPE	0.789 (0.631–0.902)	0.667	0.880

### Construction and validation of joint models

**Fig. 5:**
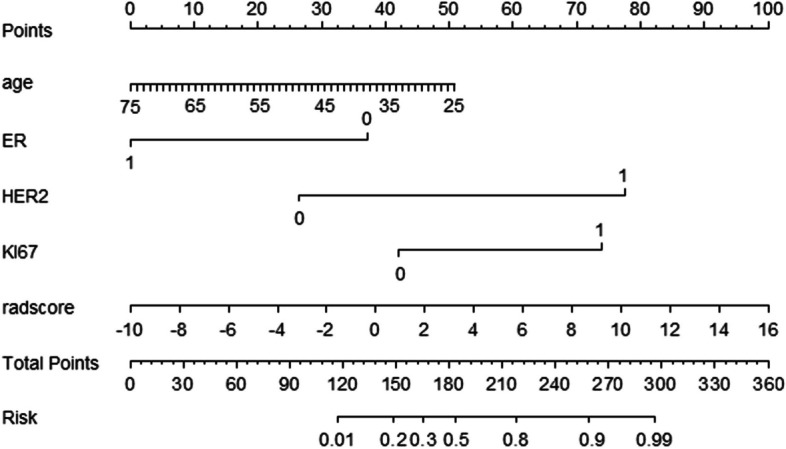
Nomogram constructed based on independent predictive factors

**Fig. 6:**
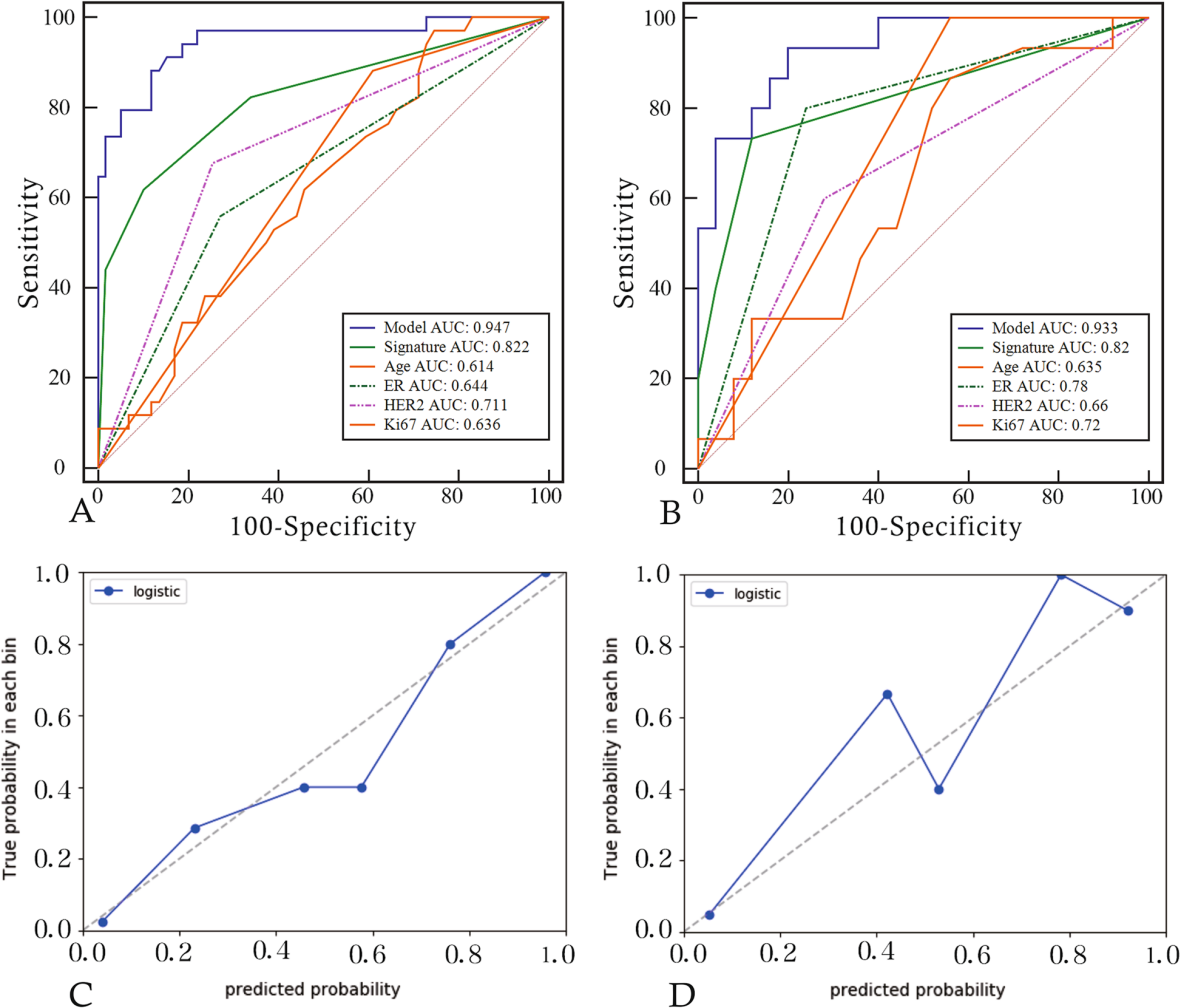
**A** and **B** show the diagnostic performance of the joint model and each independent predictor in the training and testing groups, respectively. **C** and **D** show the validation curves of the model in the training and testing groups, respectively. The closer the solid line is to the diagonal of the dashed line, the better the fitting of the model

**Fig. 7:**
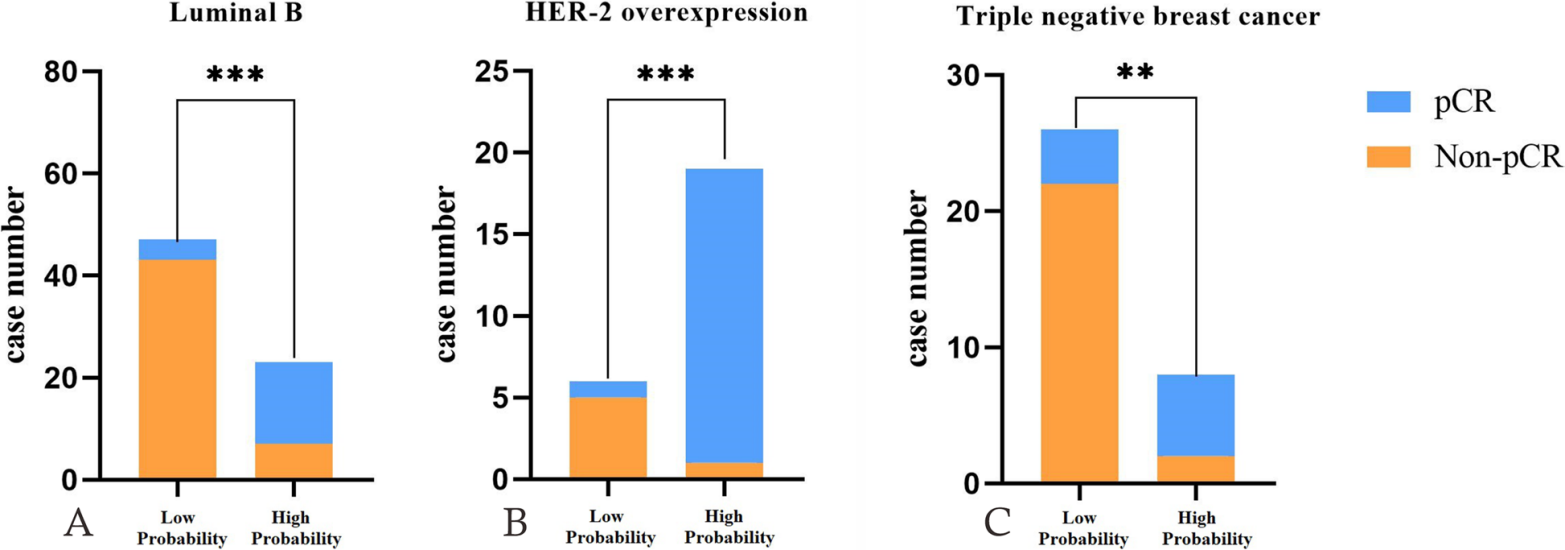
The **A**, **B** and **C** charts show the histogram of the model to distinguish high probability pCR group and low probability pCR group in luminal B, HER2 positive non luminal and triple negative

Multivariate logistic regression revealed age, ER, HER2, Ki-67, and radiomics signature as independent predictors of pCR. A joint model and a visual nomogram were subsequently developed, as depicted in Fig. 5 and Table 3. The joint model exhibited an AUC value of 0.947 and 0.933 in the training and validation sets, respectively, with a sensitivity of 0.882 and 0.933, and specificity of 0.881 and 0.8. The Delong test revealed statistically significant differences (*P* < 0.05) between the joint model and other independent predictors in both the training and validation sets, indicating improved predictive performance of the model. The validation curve demonstrated a good agreement between the predicted and actual pCR state in both sets, as illustrated in Fig. 6. Moreover, the results of the Hosmer–Lemeshow test indicated an ideal fitting of the model in both sets (*P* > 0.05). Finally, utilizing the optimal threshold of the nomogram (cut off: 0.3333), the cases were categorized into four groups based on the BCa subtypes. Except for the Luminal A without high probability group, there were significant differences between the predicted and actual pCR status in the other three groups, indicating the clinical usefulness of the model, as presented in Fig. 7.

**Table 3:** Selection of independent predictors

Variable		Univariate logistic regression		Multivariate logistic regression	
		**OR (95%CI)**	***P* value**	**OR (95%CI)**	***P* value**
Age		0.957 (0.917, 0.998)	0.042^*^	0.92(0.851, 0.994)	0.035^*^
Juejing		1.943 (0.825,4.574)	0.128	NA	NA
T		0.488 (0.172,1.383) 1.1.3831.125)	0.177	NA	NA
N		1.2 (0.431, 3.345) 0.859)	0.727	NA	NA
ER		0.294 (0.121,0.714) 4.532)	0.007^*^	0.087(0.016, 0.465)	0.004^*^
PR		0.333 (0.139,0.797) 1.426)	0.014	NA	NA
HER2		6.133 (2.427,15.501) 1.199)	< 0.001^*^	13.014(2.603, 65.07)	0.002^*^
KI67		4.792 (1.491,15.394) 3.480)	0.009^*^	1.789(0.114, 28.114) 3182.065)	0.040^*^
BI-RAD	0 vs 1	0.772 (0.079,7.532)	0.078	NA	NA
	0 vs 2	0.678 (0.265,1.736)	0.418	NA	NA
RADSCORE		2.362 (0.236,23.578)	< 0.001^*^	2.472(0.476,12.827)	< 0.001^*^

*indicates statistical significant difference

## Discussion

In this study, radiomics signature based on intratumoral regions demonstrated excellent performance in predicting PCR, which confirms previous studies. Moreover, adding the BPE region to the intratumoral region increased the predictive performance of the model for PCR. This indicates that the tumor growth environment represented by BPE impacts the efficacy of neoadjuvant chemotherapy. In addition, a joint model based on intratumoral, BPE, and clinical features provides the best prediction of pCR. These results may pave the way for comprehensive, non-invasive evaluation of individualized treatment of breast cancer [17].

This study also showed that ER, HER2 and Ki-67 were included in the construction of the model as independent predictors, which indicated that these characteristics played an essential role in the efficacy evaluation of NACT for BCa. In fact, previous studies have already established the relationship between these characteristics and neoadjuvant radiotherapy and chemotherapy. ER-positive BCa, for instance, is known to respond better to these treatments, whereas HER2-positive BCa has a worse prognosis and is highly sensitive to targeted therapy [18, 19], radiotherapy, and chemotherapy [20, 21]. Moreover, Ki-67 can reflect the sensitivity of tumors to treatment, and a significant decrease in its level can significantly improve prognosis [22]. Taking into account the findings of this study, it is advisable to consider the expression of ER, HER2, and Ki-67 when formulating individualized treatment plans for BCa patients. The study also identified age as an independent predictor in building the model, which is consistent with the findings of Li et al. [23]. They used imaging characteristics of 18F-FDG PET/CT images to predict pCR and found that the addition of age greatly improved the model’s prediction performance. However, some studies have shown that age may be unrelated to the overall pCR probability. Nonetheless, this study partially supports the conclusion that younger women with triple-negative BCa have a higher probability of pCR [24].

Based on comparisons with similar studies, Pilippo et al. used a combination of clinical/biological and radiomics features of MRI to predict pCR, with the AUC (95% CI) of the radiomics model being 0.64. This is lower than the AUC value of our radiomics signature (training set: 0.822, validation set: 0.820). The AUC of the clinical/biological–radiomics model was 0.83 [25], and our results were better (training set: 0.947, validation set: 0.933). However, incorporating BPE radiomics features in this study may be the reason for efficiency differences. BPE has become an important area of BCa research [12], and similar studies have confirmed that BPE is an important predictor of neoadjuvant chemotherapy response. R Rella’s research shows that early reduction of BPE during NAC may be an early predictor of loss of tumor response, showing potential as an imaging biomarker of treatment response [26]. In this study, we used radiomics methods to demonstrate the role of BPE in neoadjuvant chemotherapy response. BPE is a non-tumor tissue that provides a suitable environment for tumor cell growth. Therefore, BPE represents the growth environment of tumors [27] and is associated with the risk of future second cancer [28]. The results of this study further suggest that BPE may be a predictive factor for NACT response and overall BCa treatment outcomes. Therefore, this suggests that the tumor growth environment may be related to the efficacy of neoadjuvant chemotherapy.

Several studies have investigated the utility of MRI in predicting pCR before NACT. Braman et al. studied the intratumoral and peritumoral radiomics features of 117 patients and developed a Bayesian classifier with a maximum AUC of 0.93 [9], which is similar to the results of this study. However, without using Bayes classifiers, their research yielded an AUC of only 0.78. This also demonstrates the superiority of machine learning in building models, and our research further confirms the above conclusion that the efficiency of using decision trees to build models is significantly better than using traditional logistic regression models. In fact, machine learning has been widely applied to evaluate the efficacy of NACT on BCa [29–31]. In addition, the diagnostic performance of the radiomics signature based on decision tree construction in this study is significantly higher than that of the SVM-based signature constructed by Cain EH et al. (AUC: 0.707) [32]. This may be attributed to differences in the tissue regions used to obtain radiomic features. Although previous studies have demonstrated the correlation between peritumoral radiomics and pCR [33], this study did not include peritumoral tissue features in model construction. This may be because the information in the peritumoral region may not be sufficient to express the results of NACT when compared with the high heterogeneity of intratumoral and the wide range of BPE regions. Further studies are warranted to investigate the biological significance of radiomics features and to develop more accurate prediction models for personalized BCa treatment.

There are still limitations in this study. Firstly, this study is a single-center retrospective study. Due to sample size limitations, the promotion and application of the model require further validation from multiple centers and a more considerable amount of imaging data. Secondly, due to retrospective design, consistency in pathological evaluation cannot be checked. Although it is difficult to ensure that the pathological pCR status of each patient is correct, the pathological pCR status used for training and validation models is reliable. Finally, the predictive model based on radiomics has limited interpretability, which may need to explain the model’s decision-making process better. In the future, we will further study the biological significance of radiomics features to apply them to better clinical decision-making.

In summary, this study using the radiomics method demonstrated that BPE provides some value in predicting the efficacy of neoadjuvant chemotherapy PCR, thereby revealing the potential impact of tumor growth environment on the efficacy of neoadjuvant chemotherapy. This provides new insights for exploring the biological mechanisms of tumor behavior after NACT in the future.

### Supplementary Information

**Additional file 1: Table S1.** MRI parameters of each sequence. **Fig. S1.** Process of heatmap for dimension reduction in features. A and B The results of dimensionality reduction by correlation between features and actual clinical outcomes. C and D The result of dimensionality reduction using the correlation method between feature and feature. E and F The result of dimensionality reduction using GBDT. **Table S2.** Details of remaining features by multivariate logistic regression analysis. **Table S3.** The information of radiomics features. **Table S4.** The detailed information of remaining radiomics features.

## Data Availability

The datasets generated during and/or analysed during the current study are available from the corresponding author on reasonable request.

## Abbreviations

BCa: Breast cancer
pCR: Pathological complete response
BPE: Background parenchymal enhancement
NACT: Neoadjuvant chemotherapy
MRI: Magnetic Resonance Imaging
T1WI: T1-weighted images
T1 + C: Enhanced T1-weighted images
ROC: Receiver operating characteristic
AUC: Area under the curve
T: Tumor
N: Node
ER: Estrogen receptor
PR: Progesterone receptor
HER-2: Human epidermal growth factor receptor-2
PACS: Picture archiving and communication systems
VOI: Volume of interest
ITK: The insight segmentation and registration toolkit
SPM: Statistical Parametric Mapping
GLCM: Gray Level Concurrence Matrix
GLRLM: Gray-Level Run-Length Matrix
GLSZM: Gray-level size zone matrix
GLDM: Gray level co-occurrence matrix
CC: Correlation coefficient
mRMR: Maximum relevance and minimum redundancy
GBDT: Gradient Boosted Decision Number
PET/CT: Positron emission tomography-computed tomography
18F-FDG: Fluorine‑18 fludeoxyglucose
DCE-MRI: Dynamic Contrast Enhanced Magnetic Resonance Imaging
T1 + C: Enhanced T1-weighted sequences
