# Supplementary material for: Prediction of neoadjuvant chemotherapy pathological complete response for breast cancer based on radiomics nomogram of intratumoral and derived tissue
Source: BMC Med Imaging. 2024 Jan 20;24:22. doi: 10.1186/s12880-024-01198-4 (PMC10800060; doi:10.1186/s12880-024-01198-4)
Supplement: Supplementary file 1 — Additional file 1: Table S1. MRI parameters of each sequence. Fig. S1. Process of heatmap for dimension reduction in features. A and B The results of dimensionality reduction by correlation between features and actual clinical outcomes. C and D The result of dimensionality reduction using the correlation method between feature and feature. E and F The result of dimensionality reduction using GBDT. Table S2. Details of remaining features by multivariate logistic regression analysis. Table S3. The information of radiomics features. Table S4. The detailed information of remaining radiomics features. [file 12880_2024_1198_MOESM1_ESM.docx]

**Supplementary Materials**

**1. Definition of Miller-Payne grade system**

Gread 1: No change or some alteration to individual malignant cells but no reduction in overall cellularit.

Gread 2: A minor loss of tumour cells but overall cellularity still high, and up to 30% loss.

Gread 3: Between an estimated 30% and 90% reduction in tumour cell.

Gread 4: A marked disappearance of tumour cells such that only small clusters or widely dispersed individual cells remain, and more than 90% loss of tumour cells.

Gread 5: No malignant cells identiﬁable in sections from the site of the tumour, only vascular fibroelastotic stroma remains often containing macrophages. However, ductal carcinoma in situ(DCIS) may be present.

**2.** **MRI scan**

All breast MRI scans were performed in a local hospital on a 1.5T MRI scanner (Skyra; Siemens Healthineers). The routine sequences included T2-weighted imaging (T2WI), T1-weighted imaging (T1WI), diffusion-weighted imaging (DWI), and dynamic contrast-enhanced (DCE). We used post-processing workstation to acquire subtraction images. The detailed parameters for each sequence were illustrated in **Table S1**.

**Table S1.** MRI parameters of each sequence

| **Scanner** | **Sequence** | **Orientation** | **TR**  **(ms)** | **TE**  **(ms)** | **FOV**  **(mm^2^)** | **Thickness**  **(mm)** | **Interslice gap**  **(mm)** | **Matrix** |
| --- | --- | --- | --- | --- | --- | --- | --- | --- |
| SIEMENS 3.0T  (Skyra) | T_2_WI | Sagittal | 6060 | 90 | 180×180 | 3 | 0.6 | 320×224 |
|  | T_2_WI | Axial | 4790 | 134 | 200×200 | 3 | 0.6 | 384×451 |
|  | T_1_WI | Axial | 662 | 9.6 | 180×180 | 3 | 0.6 | 320×224 |
|  | DWI | Axial | 7330 | 56.0 | 200×200 | 3 | 0.8 | 112×100 |
|  | T_1_CE | Axial | 616 | 9.6 | 180×180 | 3 | 0.6 | 320×224 |

Note. TR, repetition time; TE, echo time; FOV, field of view.

**3. Image preprocessing and segmentation**

Image preprocessing including resample, intensity normalization and gray-level discretization were performed with the Pyradiomics program. To be more specific, image preprocessing was performed by resampling the images with a resolution of 1×1×1 mm3 through the linear interpolation method and by discretizing and normalizing the image gray level to order 32. Extracted texture features were standardized, which removed the unit limits of the data of each feature and converted it into a dimensionless pure value. This allowed the indexes of different units or orders to be compared and weighted. We used a z-score normalization to make the image intensities fit a standard normal distribution with $\mu\text{=0}$ and $\sigma\text{=1}$, where $\mu$ is the mean value of the images, and $\sigma$ is the standard deviation. The normalized values (also called z-scores) of the image intensities (x) were calculated as follows:

$$z=\frac{x-\mu}{\sigma}$$

Image segmentation program is as follows. Firstly, the SPM toolkit in Matlab software was used to rigorously register the images of T1WI, enhanced third-phase T1-weighted (T1+C) sequences and dynamic contrast-enhanced subtraction images in order to reduce the potential influence of the parameters of a scanning scheme. After that, the standardized T1WI images were imported into the ITK software to manually segment the entire tumor layer by layer and to determine the volume of interest (VOI). Since the four sequences have been rigorously registered, tumor VOI obtained from T1WI can be applied directly to other sequences. All cases undertook the same VOI segmentation method.

**4. Details of Dimension reduction**

First, The minimum redundancy maximum relevance (mRMR) algorithm was used to extract robust features from the dataset. The aim of the minimum redundancy process ensured that the selected features had minimal redundancy among the other features. At the same time, the maximum relevance procedure was to select features having the maximum correlation with the neoadjuvant chemotherapy pCR of breast cancer， and we selected features with correlation coefficients greater than 0.8 and 0.1 as high correlation and low redundancy features, respectively. Then, the mRMR method was used to obtain an optimal feature set with a high correlation and low redundancy.

Finally, the gradient boosting decision tree (GBDT) algorithm was used to reduce the dimension of the remaining features. GBDT is an algorithm that classifies or regresses data by the linear combination of basis functions and reduces the residual generated in the training process. In this study, 61 features were obtained from the GBDT procedur. **Fig. S1** shows the dimension reduction process. Then multivariate logistic regression was performed on these features, and seven remained. See **Table S2** for details.


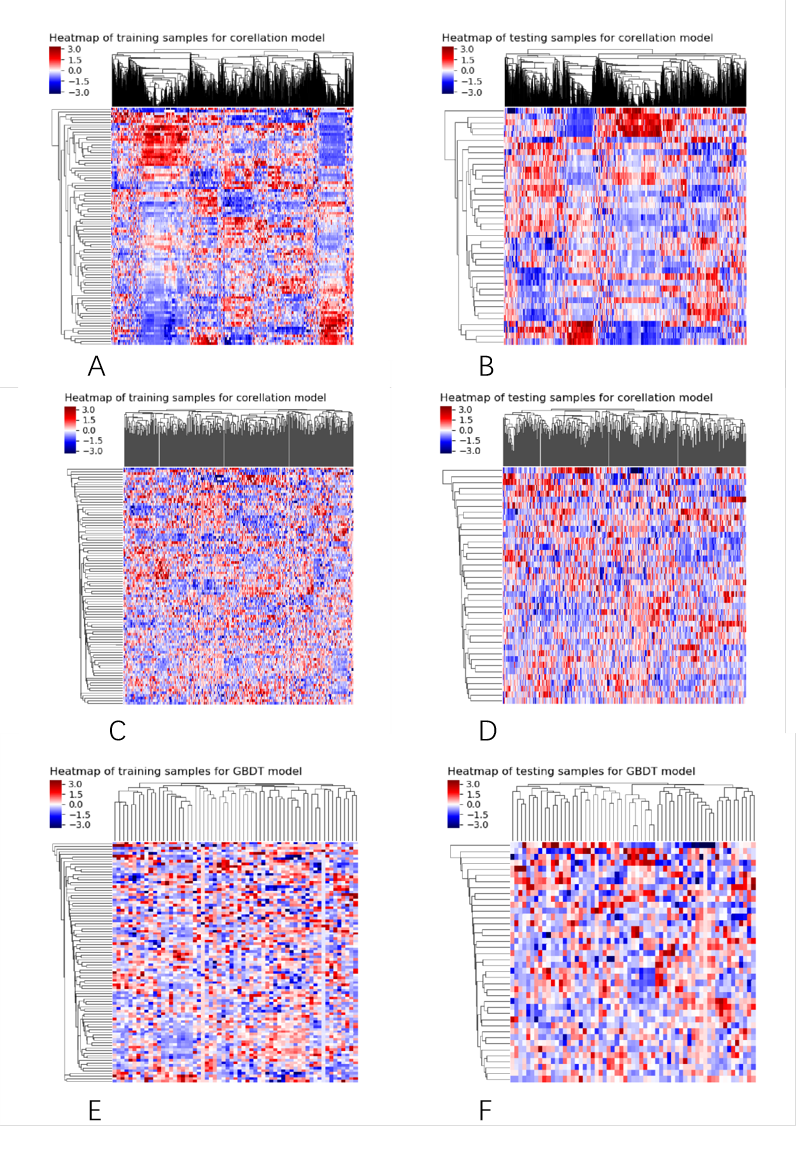


**Fig. S1**. Process of heatmap for dimension reduction in features. A and B The results of dimensionality reduction by correlation between features and actual clinical outcomes. C and D The result of dimensionality reduction using the correlation method between feature and feature. E and F The result of dimensionality reduction using GBDT.

**Table S2** details of remaining features by multivariate logistic regression analysis

| feature | OR | 0.025 | 0.975 | P_value |
| --- | --- | --- | --- | --- |
| const | 0.383 | 0.199 | 0.738 | nan |
| original_firstorder_Minimum_JY1 | 2.523 | 1.271 | 5.008 | 0.008 |
| wavelet-LLH_firstorder_Kurtosis_ZQ3 | 0.304 | 0.132 | 0.697 | 0.005 |
| log-sigma-4-0-mm-3D_gldm_LargeDependenceLowGrayLevelEmphasis_JY1 | 5.026 | 1.852 | 13.64 | 0.002 |
| wavelet-HHL_glrlm_ShortRunEmphasis_ZQ1 | 3.936 | 1.553 | 9.977 | 0.004 |
| log-sigma-2-0-mm-3D_glszm_LargeAreaLowGrayLevelEmphasis_ZQ3 | 2.607 | 1.317 | 5.163 | 0.006 |
| wavelet-LHH_glcm_ClusterShade_T11 | 3.89 | 1.326 | 11.409 | 0.013 |
| wavelet-HHH_firstorder_Kurtosis_T11 | 1.894 | 1.036 | 3.461 | 0.038 |

**Table S3.**The information of radiomics features

| Feature Groups (N) | Feature names | Feature Groups (N) | Feature names |
| --- | --- | --- | --- |
|  | firstorder_10Percentile |  | glszm_GrayLevelNonUniformity |
|  | firstorder_90Percentile |  | glszm_GrayLevelNonUniformityNormalized |
|  | firstorder_Energy |  | glszm_GrayLevelVariance |
|  | firstorder_Entropy |  | glszm_HighGrayLevelZoneEmphasis |
|  | firstorder_InterquartileRange |  | glszm_LargeAreaEmphasis |
|  | firstorder_Kurtosis |  | glszm_LargeAreaHighGrayLevelEmphasis |
|  | firstorder_Maximum |  | glszm_LargeAreaLowGrayLevelEmphasis |
| First-order features (N = 18) | firstorder_MeanAbsoluteDeviation | GLSZM texture features | glszm_LowGrayLevelZoneEmphasis |
|  | firstorder_Mean | (N = 16) | glszm_SizeZoneNonUniformity |
|  | firstorder_Median |  | glszm_SizeZoneNonUniformityNormalized |
|  | firstorder_Minimum |  | glszm_SmallAreaEmphasis |
|  | firstorder_Range |  | glszm_SmallAreaHighGrayLevelEmphasis |
|  | firstorder_RobustMeanAbsoluteDeviation |  | glszm_SmallAreaLowGrayLevelEmphasis |
|  | firstorder_RootMeanSquared |  | glszm_ZoneEntropy |
|  | firstorder_Skewness |  | glszm_ZonePercentage |
|  | firstorder_TotalEnergy |  | glszm_ZoneVariance |
|  | firstorder_Uniformity |  |  |
|  | glrlm_GrayLevelNonUniformity |  | glcm_Autocorrelation |
|  | glrlm_GrayLevelNonUniformityNormalized |  | glcm_ClusterProminence |
|  | glrlm_GrayLevelVariance |  | glcm_ClusterShade |
|  | glrlm_HighGrayLevelRunEmphasis |  | glcm_ClusterTendency |
|  | glrlm_LongRunEmphasis |  | glcm_Contrast |
| GLRLM texture features | glrlm_LongRunHighGrayLevelEmphasis | GLCM texture features | glcm_Correlation |
| (N = 16) | glrlm_LongRunLowGrayLevelEmphasis | (N = 24) | glcm_DifferenceAverage |
|  | glrlm_LowGrayLevelRunEmphasis |  | glcm_DifferenceEntropy |
|  | glrlm_RunEntropy |  | glcm_DifferenceVariance |
|  | glrlm_RunLengthNonUniformity |  | glcm_Id |
|  | glrlm_RunLengthNonUniformityNormalized |  | glcm_Idm |
|  | glrlm_RunPercentage_T2 |  | glcm_Idmn |
|  | glrlm_RunVariance |  | glcm_Idn |
|  | glrlm_ShortRunEmphasis |  | glcm_Imc1 |
|  | glrlm_ShortRunHighGrayLevelEmphasis |  | glcm_Imc2 |
|  | glrlm_ShortRunLowGrayLevelEmphasis |  | glcm_InverseVariance |
|  |  |  | glcm_JointAverage |
|  |  |  | glcm_JointEnergy |
|  |  |  | glcm_JointEntropy |
|  |  |  | glcm_MCC |
|  |  |  | glcm_MaximumProbability |
|  |  |  | glcm_SumAverage |
|  |  |  | glcm_SumEntropy |
|  |  |  | glcm_SumSquares |
|  | gldm_DependenceEntropy |  | ngtdm_Busyness |
|  | gldm_DependenceNonUniformity |  | ngtdm_Coarseness |
| GLDM texture features | gldm_DependenceNonUniformityNormalized | NGTDM texture features | ngtdm_Complexity |
| (N = 14) | gldm_DependenceVariance | (N = 5) | ngtdm_Contrast |
|  | gldm_GrayLevelNonUniformity |  | ngtdm_Strength |
|  | gldm_GrayLevelVariance |  |  |
|  | gldm_HighGrayLevelEmphasis |  |  |
|  | gldm_LargeDependenceEmphasis |  |  |
|  | gldm_LargeDependenceHighGrayLevelEmphasis |  |  |
|  | gldm_LargeDependenceLowGrayLevelEmphasis |  |  |
|  | gldm_LowGrayLevelEmphasis |  |  |
|  | gldm_SmallDependenceEmphasis |  |  |
|  | gldm_SmallDependenceHighGrayLevelEmphasis |  |  |
|  | gldm_SmallDependenceLowGrayLevelEmphasis |  |  |
| Los features (N = 186) | Log-sigma-1.0，2.0_* (N =186) |  |  |

Note: GLCM, Gray-level co-occurrence matrices; GLRLM, Gray-level run length matrix; GLSZM, Gray-level size zone matrix; GLDM, Gray-level dependence matrix. *The abbreviated representation of feature types

**Table S4.** The detailed information of remaining radiomics features

| **Sequence** | **Feature** | **Category** |
| --- | --- | --- |
| T_1_WI (n=11) | wavelet-LHH_glcm_ClusterShade_T11 | GLCM |
|  | wavelet-LHH_glcm_ClusterShade_T11 | GLCM |
| T_1_WI+C (n=11) | wavelet-HHL_glrlm_ShortRunEmphasis_ZQ1 | GLRLM |
|  | wavelet-LLH_firstorder_Kurtosis_ZQ3 | First-order |
|  | log-sigma-2-0-mm-3D_glszm_LargeAreaLowGrayLevelEmphasis_ZQ3 | GLSZM |
| T1jy (n=10) | original_firstorder_Minimum_JY1 | First-order |
|  | log-sigma-4-0-mm-3D_gldm_LargeDependenceLowGrayLevelEmphasis_JY1 | GLDM |

Note. Rad-score of the fusion model can be calculated by intercept and their respective coefficients. intercept = -1.515.

**5. Machine Learning Details**

**Decision tree (DT)**

A C5.0 decision tree based classification method was used in the analysis. C5.0 function of the “C50” package was used for creating classification trees with default parameter tuning under caret interface.
